# Liquid Biopsy in Cervical Cancer: Hopes and Pitfalls

**DOI:** 10.3390/cancers13163968

**Published:** 2021-08-05

**Authors:** Paola Cafforio, Raffaele Palmirotta, Domenica Lovero, Ettore Cicinelli, Gennaro Cormio, Erica Silvestris, Camillo Porta, Stella D’Oronzo

**Affiliations:** 1 Department of Biomedical Sciences and Human Oncology, University of Bari Aldo Moro, 70124 Bari, Italy; paola.cafforio@uniba.it (P.C.); camillo.porta@uniba.it (C.P.); 2 Interdisciplinary Department of Medicine, School of Medicine, University of Bari Aldo Moro, 70124 Bari, Italy; raffaele.palmirotta@uniba.it; 3 MASMEC Biomed—MASMEC S.p.A. Division, Modugno, 70026 Bari, Italy; dom.lovero@gmail.com; 4 Obstetrics and Gynecology Unit, Department of Biomedical Sciences and Human Oncology, University of Bari Aldo Moro, 70124 Bari, Italy; ettore.cicinelli@uniba.it (E.C.); gennaro.cormio@uniba.it (G.C.); 5 Gynecologic Oncology Unit, IRCCS Istituto Tumori “Giovanni Paolo II”, 70124 Bari, Italy; ericasilvestris85@gmail.com; 6 Division of Medical Oncology, A.O.U. Consorziale Policlinico di Bari, 70124 Bari, Italy

**Keywords:** cervical cancer, liquid biopsy, circulating tumor cells, circulating tumor DNA, circulating cell-free RNA, exosomes

## Abstract

**Simple Summary:**

Cervical cancer is the fourth most common cancer in women worldwide, and its incidence is variably distributed between developed and less-resourced countries, in which socio-economic issues and religious beliefs often limit the widespread diffusion and the access to screening campaigns. In the “liquid biopsy” era, the application of non-invasive and repeatable techniques to the identification of diagnostic, prognostic, and predictive biomarkers might facilitate the management of this disease and, hopefully, improve its outcome. The purpose of this review is to explore the progress status of liquid biopsy in cervical cancer patients. Several methods are described, which include the analysis of circulating tumor cells, the search for pathogenic mutations on circulating tumor DNA, as well as the identification of circulating RNAs, focusing on their potential clinical applications and current limitations.

**Abstract:**

Cervical cancer (CC) is the fourth most common cancer in women worldwide, with about 90% of cancer-related deaths occurring in developing countries. The geographical influence on disease evolution reflects differences in the prevalence of human papilloma virus (HPV) infection, which is the main cause of CC, as well as in the access and quality of services for CC prevention and diagnosis. At present, the most diffused screening and diagnostic tools for CC are Papanicolaou test and the more sensitive HPV-DNA test, even if both methods require gynecological practices whose acceptance relies on the woman’s cultural and religious background. An alternative (or complimentary) tool for CC screening, diagnosis, and follow-up might be represented by liquid biopsy. Here, we summarize the main methodologies developed in this context, including circulating tumor cell detection and isolation, cell tumor DNA sequencing, coding and non-coding RNA detection, and exosomal miRNA identification. Moreover, the pros and cons of each method are discussed, and their potential applications in diagnosis and prognosis of CC, as well as their role in treatment monitoring, are explored. In conclusion, it is evident that despite many advances obtained in this field, further effort is needed to validate and standardize the proposed methodologies before any clinical use.

## 1. Introduction

Despite the development of effective primary and secondary prevention strategies [1], cervical cancer (CC) is still a major public health problem for middle-aged women, especially in countries with fewer resources. In 2018, it was the fourth most common cancer in women worldwide, after breast, colorectal, and lung malignancies, with about 90% of cancer-related deaths occurring in developing parts of the world [2].

Such a geographical influence on disease evolution reflects differences in the prevalence of human papilloma virus (HPV) infection, which is the main cause of CC, as well as in the access and quality of services for CC prevention and diagnosis [3].

During their lifetimes, more than 80% of women and more than 90% of men are expected to be infected with HPV, generally before the age of 45 [4]. Even if the vast majority of these infections resolve spontaneously within a couple of years, others may persist and lead to slow and progressive changes within the cervix that can ultimately result in cancer development [5].

The Papanicolaou (Pap) test has been for decades the standard method for CC screening, but its relatively low sensitivity (about 50%) and reproducibility [6] have led to the incorporation of HPV-DNA test into screening programs, which has been shown to provide 60–70% greater protection against invasive CC, compared to Pap-test alone [7].

However, several studies have shown that in low- and middle-income countries, socioeconomic, cultural, and ethical issues adversely affect the quality of both prevention and treatment of gynecological cancers [8]. In particular, cultural and religious beliefs in some regions of Africa, Middle East, and Asia induce patients to underestimate the severity of these diseases and, in some cases, preclude any diagnostic and therapeutic approaches [8,9,10,11]. Moreover, barriers are commonly raised by the fear of social and familial disapproval [9] towards gynecological practices. It is of note that similar issues are often encountered within ethnic groups immigrating to Western countries, in whom a higher incidence of gynecological malignancies is observed compared to the native population [12,13].

These observations suggest that a “liquid biopsy” based approach may theoretically represent a valid additional (or alternative) model for CC screening, diagnosis, and follow-up [14], besides giving potential prognostic and predictive information.

Indeed, the considerable biotechnological advances achieved in recent years, thanks to the advent of next-generation sequencing (NGS), digital-PCR (dPCR), and other high throughput “omics” techniques, have led to the possibility of studying minimal amounts of RNA or DNA from tumor cells [15,16].

Such a high analytical sensitivity has been successfully applied to the research and identification of tumor cells within body fluids, in most cases, peripheral venous blood.

At present, the most popular liquid biopsy approaches consist of the detection and isolation of circulating tumor DNA (ctDNA) and circulating tumor cells (CTCs).

ctDNA represents a fraction of circulating cell-free DNA (ccfDNA), namely the cell-free circulating nucleic acid fragments normally present in plasma and serum as a consequence of both active release by lymphocytes and cell lysis, which is specifically attributable to the lysis of tumor cells in the bloodstream [17]. The analysis of ctDNA can provide information that is in some agreement with that obtained from the molecular screening of tumor tissue biopsies, enabling the identification of somatic gene mutations, polymorphic sequence variants, or gene fusions that are useful for diagnosis, definition of patient prognosis, and risk of disease recurrence, or ideally, act as therapeutic targets [16,18].

CTCs are poured into the bloodstream from primary or secondary tumor sites, and despite their very small number with a frequency of approximately 1–10 tumor cells in 10^6^–10^8^ white blood cells, they are potentially able to give origin to distant metastases [18]. The identification and isolation of CTCs, especially in patients with early-stage disease, are closely related to the sensitivity and specificity of the isolation methods, usually consisting of sophisticated procedures including preliminary enrichment and subsequent isolation steps [16,19]. Numerous studies on breast, gastrointestinal, lung, skin, and prostate malignancies have shown that a CTC count above cut-off values between 3 and 5 CTCs/7.5 mL blood, determined by using the CellSearch platform, is associated with shorter progression-free survival (PFS) and overall survival (OS) [16,20,21,22]. CTCs are also a valuable source of well-preserved nucleic acids, useful for downstream mutational and gene expression analyses.

In addition to ctDNA and CTCs, the study of circulating cell-free RNA (ccfRNA) plays a considerable role in the context of liquid biopsy, with significant diagnostic and prognostic implications. Indeed, similarly to ctDNA, ccfRNA molecules are released into the circulation under both physiological and pathological conditions [23]. In this context, there is considerable interest in miRNAs, namely small non-coding RNAs that regulate gene expression at the post-transcriptional level. miRNAs mediate intercellular communication, and alterations in their expression profile are closely correlated with the onset and/or progression of many types of cancers [24].

In addition to these methods, there are many others, even more innovative, that still require clinical validation and analytical standardization. For instance, cancer cells are known to release “extracellular vesicles” (EV) into the circulation, which carry key elements, such as DNA, messenger RNA (mRNA), microRNA, and proteins, useful to investigate tumor characteristics since its very earliest stages [16,19,25].

This review aims to describe the state of the art and the current knowledge of liquid biopsy in CC, focusing on the perspectives and limitations of the main methodologies employed, their potential diagnostic and prognostic applications, as well as their theoretical therapeutic implications.

## 2. Circulating Tumor Cells (CTCs)

Collection of CTCs represents a non-invasive, real-time means to investigate tumor heterogeneity, as well as to monitor disease evolution and response to treatment over time. Moreover, the identification of specific molecular targets on CTCs may enable us to recognize resistance mechanisms and test new potential therapeutic agents [16,20].

The strategies for detecting and isolating CTCs in CC generally rely on physical and morphological properties of the cells, as well as on the identification and quantification of HPV oncogenes and epithelial markers, by using molecular and/or immunofluorescence procedures [26,27,28]. Despite the numerous attempts made by researchers in this field, CTC identification and quantitation is still scarcely diffused in CC compared to other solid malignancies, probably due to the lack of specific tumor markers, as well as to the low number of CTCs detectable within peripheral blood samples. In this regard, the number of CTCs has been shown to vary in relation to the cancer clinical stage, ongoing therapies, and isolation methods [29].

Several groups applied nucleic acid-based methods to detect CTCs in blood (Table 1). In particular, mRNA amplification of specific markers was considered more useful than ctDNA detection, since mRNA is extremely labile and degradable in biological samples and, therefore, specifically reflects the presence of circulating viable cells, compared to ctDNA, which is more stable and can derive also from necrotic or apoptotic ones [28].

In 1997, Stenman and colleagues detected uterine cervix-derived CTCs by searching for squamous cell carcinoma antigen (SCC-Ag) via RT-PCR [30]. On the other hand, several authors detected HPV mRNA in the peripheral blood of metastatic CC patients [31] whereas, in other instances, the detection of cytokeratin 19 (CK19) mRNA was used to this purpose [32]. However, in the latter case, even if the authors detected CK19 expression in 21.4% of CC patients, compared to its absence in healthy donors, no correlation with clinical parameters or survival was found [32].

A few years later, Fehm and colleagues tried to identify disseminated tumor cells (DTCs) in the bone marrow of patients with gynecological tumors by immunohistochemical staining for pan-cytokeratin. The authors demonstrated that 26% of patients with CC presented DTCs, and this finding significantly correlated with FIGO tumor stage (*p* < 0.05) but not with other known prognostic factors [39]. Subsequently, they confirmed the same data on a greater cohort of patients, finding a correlation with primary tumor size and nodal involvement [40,41]. However, the presence of these cells in the bone marrow did not correlate with OS nor with disease-free survival (DFS) [42].

By exploiting more sensitive molecular techniques, Pfitzner proposed the digital-direct-RT-PCR for HPV16 and HPV18 viral transcripts as a useful method to detect and quantify CTCs in patients with CC [33]. In particular, in 3 out of 10 patients, they found the presence of a single CTC expressing the HPV oncogene transcript among 5 to 15 × 10^5^ peripheral blood cells, demonstrating the high sensitivity of the method.

An anecdotal report on a single CC patient described a size-based method to enrich and isolate CTCs, based on the sample filtration through a polycarbonate membrane with 8 μm pores. Interestingly, CTCs were cultured and then characterized for their viability, cytomorphological properties, and gene expression for tumor- and chemoresistance-associated genes. Moreover, also ovarian and endometrial cancer CTCs from two separate patients were analyzed, but the gene expression analysis was not able to demonstrate any differences among cancers [34].

Takakura and colleagues used an alternative method to identify CTCs by infecting nucleated cells in 23 blood samples from CC patients, at different disease stages, with a green fluorescent protein (GFP)-modified adenoviral vector, namely OBP-1101, which specifically targets cancer cells characterized by high telomerase activity [35]. Then, the cells were stained with anti-CD45, anti-pan-cytokeratin, and anti-CK19 antibodies to discriminate CTCs by residual blood components, whereas nuclei were stained by 4′,6-diamidino-2-phenylindole (DAPI). CTCs were identified, captured by automatically manipulated glass pipettes and tested for HPV gene to confirm their origin. CTC analysis revealed that these cells had lost their epithelial features (since they were cytokeratin-negative), but maintained the same HPV subtypes infecting tumor cells in the primary lesion. However, CTC count did not correlate with disease status and the method turned out to be time consuming and strictly dependent on viral infection ability. For these reasons, it was considered not applicable to a large cohort of patients and was not further developed.

On the other hand, a wider study conducted on 99 patients with locally advanced CC (FIGO IIB-IVA) who had undergone radiotherapy or chemoradiotherapy demonstrated that the combination of SCC-Ag levels and CTC count was significantly associated with DFS (HR 2.711, *p* = 0.000) [36]. Moreover, by multivariate analysis, the authors stated that CTC number, FIGO stage, and serum SCC-Ag level were independent prognostic factors for 2-year DFS and suggested this method as a new risk model to predict disease progression. Interestingly, CTCs were identified by a new methodology including negative enrichment and detection of chromosome 8 (CEP8) hyperdiploid status based on immunofluorescence in situ hybridization (NEimFISH), in the absence of CD45 as a leukocyte marker.

Based on previous reports describing the role of epithelial to mesenchymal transition (EMT) in the migration and survival of CTCs, as part of the metastatic cascade [39,40,41], Pan and colleagues analyzed the phenotype of CTCs from 90 patients with FIGO I-IIA CC, which were further classified as “epithelial”, “mesenchymal”, or “mixed” [37]. CTCs were enriched by CanPatrol^TM^ technique, together with an anti-CD45 antibody, and characterized by branched DNA signal amplification for epithelial (EPCAM, CK8) and mesenchymal (Vimentin, TWIST) markers. In this study and in agreement with previous findings, higher clinical stage, the presence of lymph node metastases, lymphovascular and stromal invasion correlated with a higher number of mesenchymal CTCs (*p* < 0.01).

More recently, another study has evaluated the prognostic significance of CTCs in patients with CC and analyzed the relationship of this parameter with demographics and clinical characteristics. In particular, 107 blood samples, collected after radiotherapy or during concurrent cisplatin-based chemotherapy, were analyzed for their CTC content [29] by applying a preliminary negative enrichment and the previously described NEimFISH method [36]. Patients with at least one CTC had significantly shorter PFS (median PFS 39.8 months; 95% CI: 34.6–45.0 months) than CTC-negative ones (median PFS 44.8 months; 95% CI: 40.6–49.0 months; *p* = 0.018) over a 2-year follow-up and, based on these results, the authors proposed the CTC count as an independent prognostic factor in CC.

For the first time, a phase III randomized clinical trial leading to the regulatory approval of bevacizumab in recurrent/metastatic CC investigated the predictive/prognostic role of CTC count [38]. The total population enrolled in the study comprised 452 patients, randomized to receive different taxane-containing therapies with or without bevacizumab. CTCs were analyzed in 176 patients at baseline and at 36 days post-cycle 1 by CellSearch using anti-epithelial cell adhesion molecule (EPCAM) microbeads. Finally, CTCs were isolated and, subsequently, characterized by immunofluorescence microscope as anti-cytokeratin positive/anti-CD45 negative cells. The median CTC count at baseline was 7 cells/7.5 mL blood versus 4 cells/7.5 mL post-cycle 1, regardless of treatment arm. Patients with low CTC count at baseline did not significantly benefit from bevacizumab addition in terms of OS (15.8 versus 17.1 months) and experienced a median OS similar to patients with high CTC count who did not receive bevacizumab (16.2 months). In a similar fashion, the PFS of women with low CTC count was not significantly modified by bevacizumab addition (median PFS 7.3 versus 6.2 months; HR 0.95; 95% CI, 0.58–1.55), whereas this outcome was significantly improved in patients with high CTC count at baseline who received bevacizumab compared to controls (median PFS 10.8 vs. 6.9 months, HR 0.59; 95% CI, 0.36–0.96). This effect was more pronounced in subjects receiving cisplatin-paclitaxel chemotherapy backbone, in whom the median PFS with and without bevacizumab was 14.6 versus 6.4 months (HR 0.26; 95% CI, 0.12–0.55), respectively. Based on these data, the authors proposed CTC count as a predictive biomarker to guide treatment selection in these patients [38].

Although the identification and counting of CTCs in CC is considered an interesting topic worthy of further investigation, the literature evidence is still contradictory, to date. First of all, the methods used to detect CTCs are quite variable and probably each of them underestimates the real amount of CTCs in a blood sample. Furthermore, there is no clinical homogeneity in the patient populations enrolled in the different studies, and therefore, it is difficult to draw definitive conclusions about CTC significance in prognosis or prediction of response to therapy, whereas there is a lack of studies investigating the putative diagnostic meaning of CTCs in this setting. In addition, some markers used to identify CTCs are lost during the EMT process, following extravasation and adaptation to the microenvironment. Therefore, a consensus would be necessary to obtain more reliable and reproducible data on the usefulness of CTCs in this setting. Moreover, the application of deep sequencing technologies to CTCs might provide additional information on their molecular heterogeneity and consequent potential clinical implications.

## 3. Circulating Cell-Free DNA (ccfDNA)

Cancers are well known to shed ctDNA into the bloodstream, although the exact mechanisms for ctDNA release are still unclear [16,42]. Moreover, the amount of ctDNA derived from tumor cells depends on several factors, i.e., the tumor volume or turnover rate [43], and represents only a small fraction of the whole plasma ccfDNA [14,16]. This highlights the need for extremely sensitive detection methods for ctDNA to be applied in the clinical practice, but to date, little is known about the clinical implications of ctDNA assessment in gynecologic malignancies and, more specifically, in CC [44].

Preliminary reports based on the dosage of ctDNA levels in patients with CC versus healthy controls showed that plasma ctDNA content was significantly higher in the former and associated with International Federation of Gynecology and Obstetrics (FIGO) tumor stage (*p* < 0.05), histological grade, infiltration depth, and presence of lymphatic metastases [45,46].

Based on previous studies that extensively characterized the mutational spectrum of CC [47], some authors applied targeted NGS panels or dPCR to ctDNA detection to identify gene variants potentially useful for diagnosis, prognosis, or evaluation of response to therapy (Table 2).

One of the first analyses performed by dPCR on plasma samples from Chinese women with primary invasive CC allowed the identification of *PIK3CA* mutations (i.e., p.E542K and p.E545K) in 26 out of 117 (22.2%) patients. The presence of *PIK3CA* alterations significantly correlated with high pathological grade (*p* < 0.001) and large tumor size (*p* < 0.05), as well as with decreased disease-free survival (DFS) and OS (*p* < 0.05 in both instances) [48], in agreement with previous studies describing the role of *PIK3CA* alterations in cervical tumorigenesis [52].

Later on, Tian and coworkers, by employing a targeted NGS analysis of 48 cancer-relevant genes on ctDNA samples from 57 Chinese CC patients, identified a high frequency of pathogenic mutations in *KDR* (35.1%), *ALK* (33.3%), *EGFR* (33.3%), and *ATM* (31.6%), while *PDGFRA*, *CSF1R*, *ERBB2*, *HNF1A*, *NOTCH1*, *TP53*, *APC*, *KRAS,* and *PTPN11* mutations had a frequency just over 20%. In order to correlate these data with clinical outcome, the authors used an algorithm to estimate the allele fraction deviation (AFD), obtained by calculating for each patient the deviance between the mutant allele fraction (MAF) found in the ctDNA and the one found in matched white blood cells. Despite the small sample size, which limited statistical considerations, the study highlighted that AFD value tended to decrease in CC patients after treatment (surgery, chemotherapy, radiotherapy, or multimodal approaches) in parallel with tumor size while, when remaining steadily high, it did correlate with disease progression and metastasis onset. In addition, in patients with low baseline AFD, a subsequent increase was suggestive of relapse [51].

In another small study, Lee and coworkers used a customized NGS panel including 51 target genes to isolate both ctDNA and CTC-DNA from the peripheral blood of 20 gynecological cancer patients (including four cases of CC) and compared the variants found in each couple of DNA samples, describing BRCA2, ERBB2, ESR1, FGFR4, PTCH1, STK11, and TSC2 as common overlapping mutated genes in the overall cohort [53].

Subsequently, the same research group focused on CC patients, collecting and analyzing the ctDNA from 24 Korean women one week prior to primary treatment by using another custom NGS panel made up of 24 genes, previously described within The Cancer Genome Atlas program [47,50]. The authors identified alterations in 18 out of 24 genes analyzed, among which *ZFHX3*, *KMT2C*, *KMT2D*, *NDS1*, *ATM,* and *RNF213* were found mutated with a frequency ranging from 27% to 83%. All subjects harbored mutational events in at least three genes, with an average of nine variants per patient, whose longitudinal monitoring was proposed by the study authors as a promising means to evaluate treatment response over time [50].

In another study, Tian and colleagues applied a wider NGS panel (including 1517 hotspot regions in 313 cancer-related genes) to the analysis of ctDNA samples from 52 disease-free and 30 metastatic or relapsed CC patients. By using this approach, the authors identified specific mutations in PIK3CA, BRAF, GNA11, FBXW7, and CDH1 genes in the ctDNA isolated from the metastatic cohort, with a 25% prevalence of *PIK3CA* alterations, while less common mutations were found in other genes (e.g., ALK, STK11, VHL, PDGFRA, HNF1A, MPL, and ABL1). Patients with less than two metastatic sites harbored a number of mutations significantly lower than those with ≥2 lesions (*p* = 0.001); moreover, a significant correlation emerged between the presence of any of the above mentioned mutations and shorter PFS (HR 2.57, 95% CI: 1.20–5.52, *p* = 0.005) and OS (HR 2.66, 95% CI: 1.20–5.87, *p* = 0.007). In addition, the authors attempted to investigate whether the identified mutations correlated with response to chemotherapy; to this purpose, a total of 23 metastatic CC patients underwent serial ctDNA analyses, and interestingly, a reduction in the number of variants over time was significantly associated with response to treatment, while the reverse was true for patients who experienced disease progression (OR 21.00, 95% CI: 2.07–131.90; *p* = 0.007) [49].

A recent work by Charo and coworkers has described the application of a 73 gene-NGS panel to the ctDNA analysis of 105 gynecologic cancer patients enrolled in the Profile Related Evidence Determining Individualized Cancer Therapy (PREDICT) clinical trial. Within the CC cohort of this study (N = 13), the authors have identified PIK3CA and TP53 gene mutations in 61.5% and 38.5% of cases, respectively, followed by *FBXW*7, *ERBB2,* and *PTEN* with frequencies slightly above 10%. Once mutational analyses were matched with clinical data, the number of mutations found in the ctDNA emerged as significantly correlated with OS (HR 1.91, *p* = 0.03) within the whole patient cohort. Moreover, treatment selection according to ctDNA analysis findings has been associated with improved OS (*p* = 0.007), confirming the potential applicability of such an approach to the clinical practice, for prognostic and predictive purposes [44].

Another recent study by Zhang and coworkers has described the largest ctDNA analysis so far, performed on over 10,000 Chinese patients and covering a broad range of cancer types, among which were 126 cases of CC. The NGS of these samples, performed with a panel covering 1020 genes, showed *PIK3CA* as the most frequently mutated gene (about 30%) followed by *MLL3*, *TP53*, *MLL2*, *EP300*, *PTEN*, *FGFR3*, *DNMT3A*, *PTCH1,* and *TERT*. Based on these results, largely overlapping those derived from CC tissue sequencing and previously reported [47], the authors have concluded in favor of the broad application of ctDNA within precision medicine programs [42].

Another line of research that is worth mentioning in a CC setting has focused on circulating HPV DNA as a potential tumor marker due to the well-established role of this virus in cervical carcinogenesis [54,55].

A noteworthy meta-analysis on this topic was performed by Gu and coworkers by examining 10 eligible studies up to March 2019, which involved 684 CC patients overall. This study, which compared different technologies as real-time PCR, methylation-specific PCR, and dPCR performed on patients’ plasma or serum, highlighted an overall 0.27 sensitivity and 0.94 specificity of these methods, demonstrating that dPCR was the most accurate in detecting HPV cDNA [55].

In a similar fashion, Cheung and coworkers [43] demonstrated this proof of concept by applying dPCR to the detection of HPV DNA fragments in the blood samples of 138 CC patients. Although limited by the small sample size, this study provided evidence that patients with a high viral load (defined as ≥20 copies per 20 μL of the reaction volume) had increased risk of recurrence (*p* = 0.03) and death (*p* = 0.007) at 5 years [43].

Moreover, a correlation between HPV DNA methylation and CC onset was described by Guerrero-Preston and coworkers, who tried to test whether a high-throughput panel of methylated viral and human genes could identify women with grade 2 or higher cervical intraepithelial neoplasia (CIN). Rather than on blood, this panel was set up on liquid-based cervical cytology samples over a prospective cohort of 107 women and also tested on urine, successfully discriminating cervical samples with pre-cancerous lesions from the negative ones [56].

In conclusion, despite the few studies investigating the correlations between ctDNA and CC, these preliminary results suggest promising perspectives of this method in the clinical monitoring, prognostic, and therapeutic evaluation of CC patients, whose development is recommended.

## 4. Total and Cell-Free Circulating RNA

### 4.1. Coding RNAs

In addition to CTCs and ctDNA, circulating RNAs have been widely investigated in CC for diagnostic and prognostic purposes (Table 3) [57].

The first studies on this topic focused on coding RNAs, namely those RNAs that are translated into proteins and act as “messengers” (mRNAs) of genetic information from the cell nucleus to the cytoplasm [58].

Several mechanisms have been proposed to explain the presence of RNAs in the bloodstream, including tumor cell necrosis, apoptosis, and active release by cancer cells [23].

With respect to mRNAs, in 1997, Pao and coworkers described the presence of transcriptional products of the HPV-16 E6 transforming gene in the whole peripheral blood of 12 patients with HPV-positive metastatic CC by applying a RT-PCR based protocol [31]. A few years later, the same authors identified both HPV-16 and HPV-18 circulating E6 mRNAs in 18 out of 35 CC patients, describing a significant correlation between the presence of such biomarkers and high-risk disease features, namely, a primary tumor diameter >4 cm (*p* = 0.03), positive pelvic lymph nodes (*p* = 0.03), and distant metastases (*p* = 0.01) [59].

In a similar fashion, the detection of total circulating epidermal growth factor receptor (EGFR) mRNA by RT-PCR was found to correlate with FIGO clinical stage of CC (*p* < 0.05); on the other hand, tumor histological features and size, patient age, and nodal status were not associated with EGFR mRNA detection [60].

A few years later, Zhang et al. attempted to isolate B cell-specific moloney murine leukemia virus integration site 1 (Bmi-1) mRNA (encoding a transcriptional modulator that participates in cell proliferation and cancer progression) in the plasma of CC patients, as well as in women with CIN and healthy controls (N = 109, 138 and 80, respectively). By applying a RT-PCR based approach, the authors described significantly higher circulating levels of this mRNA in CC patients compared to those in “CIN” and “control” groups (median values: CC = 0.129; CIN3 = 0.037; CIN2 = 0.023; CIN1 = 0.004; control = 0.003; *p* < 0.001 in all instances). Moreover, a correlation between this putative biomarker and clinical stage was proven (*p* < 0.001), while the optimal cut-off value was set at 0.057, reaching a 69.7% sensitivity and a 95.9% specificity [61]. Interestingly, Kaplan–Meier analysis further demonstrated a correlation between high circulating Bmi-1 mRNA levels and poor DFS (*p* = 0.001) and OS (*p* = 0.015) [61].

However, due to the huge amount of ribonucleases found in the serum of cancer patients [62], degradation of extracellular circulating mRNAs was frequently observed, together with potential contamination by intracellular mRNAs [63]. This limited both the reproducibility and the applicability of circulating mRNAs as cancer biomarkers, leading to the development of further, probably more reliable, RNA-based technologies that will be discussed in the next sections.

### 4.2. Non-Coding RNAs

Non-coding RNAs are functional RNA molecules that lack the capacity of protein coding but actively participate in the epigenetic regulation of gene expression. This subgroup of RNAs is made up of long non-coding RNAs (lncRNA), short interfering RNAs (siRNAs), microRNAs (miRNA), and piwi-interacting RNAs (piRNA), among others [64].

These epigenetic modulators have been shown to play key roles in cell transformation, participating in different steps of both CIN and CC development [65]. Most observations in the field of circulating RNAs applied to CC involve lncRNAs and miRNAs and will be discussed here (Table 3).

**Table 3:** Pre-clinical and clinical studies investigating the role of circulating RNAs in CC.

Type of RNA	Biomarker Name	N. of CC Patients	Putative Clinical Validity	References
**mRNA**	HPV-16 E6 mRNA HPV-18 E6 mRNA	35	Prognostic	[59]
**mRNA**	EGFR mRNA	45	Prognostic	[60]
**mRNA**	Bmi-1 mRNA	109	Diagnostic Prognostic	[61]
**lncRNAs**	HOTAIR, PVT1, AL592284.1, XLOC_000303	300	Diagnostic	[66]
**lncRNA**	AC017078.1 XLOC_011152	24	Diagnostic	[67]
**lncRNA**	lncRNA DLX6-AS1	114	Diagnostic Prognostic	[68]
**miRNA**	miR-218	90	Diagnostic Prognostic	[69]
**miRNA**	miR-20a	80	Diagnostic	[70]
**miRNAs**	miR-20a, miR-1246, miR-2392, miR-3147, miR-3162-5p, miR-4484	80	Prognostic	[71]
**miRNA**	miR-196a	105	Diagnostic Prognostic	[72]
**miRNAs**	miR-21, miR-25, miR-29a, miR-200a, miR-486-5p	213	Diagnostic	[73]
**miRNA**	miR-138	Pre-clinical study	Therapeutic	[74]
**miRNA**	miR-148b	Pre-clinical study	Therapeutic	[75]
**miRNA**	miR-425-5p	40	Diagnostic Prognostic	[76]
**miRNA**	miR-30e	Pre-clinical study	Therapeutic	[77]
**miRNA**	miR-187	60	Prognostic	[78]
		Pre-clinical study	Therapeutic	
**miRNA**	miR-138	168	Prognostic	[79]
		Pre-clinical study	Therapeutic	
**miRNA**	miR-195	Pre-clinical study	Therapeutic	[80]
**miRNA**	miR-214	Pre-clinical study	Therapeutic	[81]
**miRNA**	miR-486-5p	Pre-clinical study	Diagnostic Therapeutic	[82]
**miRNAs**	miR-17-5p, miR-32-5p, miR-409-3p, miR-454-3p	115	Diagnostic	[83]
**miRNAs + protein**	miR-25, -29a, -486-5p (+ SCC Ag)	200	Diagnostic	[84]
**exosomal miRNAs**	let-7d-3p, miR-30d-5p	63	Diagnostic	[85]
**exosomal miRNA**	miR-125a-5p	44	Diagnostic Prognostic	[86]

LncRNAs consist of more than 200 nucleotides and control tumorigenesis and metastasis processes by interacting with chromatin-modifying complexes or acting as a decoy for transcription factors and miRNAs. These molecules are easily detectable in different body fluids, including serum and plasma [67], either in free-circulating form or enclosed into small extracellular vesicles, such as apoptotic bodies or exosomes [87], whereas a fraction of lncRNAs is released in body fluids conjugated with stabilizing proteins [88].

Among free-circulating lncRNAs, HOX transcript antisense intragenic RNA (HOTAIR) has been found overexpressed in several solid malignancies, including breast, cervical, and ovarian cancer [89,90]. Indeed, preliminary in vitro studies demonstrated a significant upregulation of HOTAIR in CC cell lines, with its inhibition resulting in suppression of tumor cell proliferation and migration [91]. In 2018, Sun and coworkers described a 3.5-fold over-expression of HOTAIR in the whole blood of 300 CC patients compared with 180 healthy controls. Once this putative biomarker was combined with other three lncRNAs (i.e., PVT1, AL592284.1, and XLOC_000303), the positive and negative predictive values of this composite score reached 88% and 84%, respectively [66]. In another work by Iempridee et al., two lncRNAs (i.e., AC017078.1 and XLOC_011152) were found significantly downregulated in the serum of both early stage (I/II) and advanced (III/IV) CC patients compared to healthy controls (*p* < 0.0001 in both instances), suggesting the putative diagnostic role of these biomarkers [67].

More recently, Ding and coworkers have explored the potential application in CC of another lncRNA, namely DLX6-AS1, already known for its oncogenic role in other solid malignancies [92,93,94,95]. The authors have evaluated, via quantitative RT-PCR, its serum exosomal levels in 114 CC patients, as well as in 60 women with CIN and 110 healthy subjects, describing not only significantly higher concentrations of this marker in CC patients versus CIN and controls (*p* < 0.001 in both instances) but also a positive correlation with lymph node metastasis (*p* = 0.0071) and FIGO stage (*p* < 0.0001); in addition, exosomal lncRNA DLX6-AS1 has turned out to be an independent prognostic marker for OS (HR = 3.38, 95% CI = 1.742–6.178, *p* = 0.009) in multivariate analysis [68].

On the other hand, miRNAs are single-stranded RNAs made up of approximately 19–25 nucleotides, potentially acting as either oncogenic (oncomiR) or tumor suppressive molecules and, hence, often deregulated in patients with solid malignancies, including CC [57,96]. In particular, these RNAs are able to bind mRNAs and participate in the post-transcriptional regulation of key genes involved in cell proliferation, invasion, and migration [96]. Similarly to lncRNAs, miRNAs can be found both in the cellular compartment and extracellularly, bound to proteins or within extracellular vesicles [97].

A meta-analysis of miRNA profiles related to CC was performed by He et al., including 3922 primary tumor samples and 2099 controls; the analysis showed 63 differentially expressed miRNAs (DEmiRs) between the two groups (42 up- and 21 downregulated in CC), most of which were found to target such key oncogenic pathways as ErbB, MAP kinase, mTOR, p53, TGFβ, and Wnt [98].

Later on, several authors focused on free-circulating miRNAs for diagnostic, prognostic, or therapeutic purposes [57].

Zhao and coworkers described a significant upregulation of miR-20a in the serum of CC patients, and especially in those with lymph node metastases, compared to healthy controls (*p* = 0.004 and *p* = 0.000, respectively) [70].

Afterwards, multiparametric panels were developed to optimize the ability of miRNAs to act as CC diagnostic biomarkers. Among these, a panel made up of five serum miRNAs (i.e., miR-21, -25, -29a, -200a, and -486-5p) was identified by Jia et al. through a two-step procedure, based on preliminary genome-wide miRNA sequencing followed by quantitative PCR (qPCR) verification. The panel successfully distinguished CC patients from healthy controls, reaching a higher AUC value (0.908, 95% CI: 0.868–0.948) than those observed for any of the single miRNA-based assays (range 0.658–0.819). Moreover, at its optimal cut-off value, the specificity and the sensitivity of the test were 88.6 and 81.0%, respectively [73]. Recently, another group has further validated three of the above mentioned miRNAs (i.e., miR-25, 29a, and -486-5p) in association with a serum protein biomarker (i.e., SCC Ag), within a multi-parametric panel that is able to distinguish early-stage CC patients from healthy controls, with a sensitivity of 80.0% and specificity of 96.7% [84].

In addition to having diagnostic potential, some cell-free circulating miRNAs were found able to provide prognostic information in CC patients.

In consideration of its tumor suppressive activity described in other solid malignancies [99,100], miR-218 was the focus of a study by Yu et al., who evaluated its expression by qPCR in the serum of 90 CC patients compared to age-matched controls. Notably, the authors demonstrated not only a significant downregulation of the molecule in the former group (*p* < 0.001), as expected, but also an association of its expression with tumor stage (stage I-II: 0.425 ± 0.033; stage III-IV: 0.128 ± 0.016, *p* < 0.001) and two-year survival probability, although such a correlation did not reach statistical significance [69]. However, the putative prognostic role of miR-218 was not further investigated in prospective clinical studies.

On the other hand, miR-196a, which is known to promote tumor cell proliferation and migration [101,102], was found over-expressed in CC cell lines and tissues [101,103]. In an attempt to evaluate its putative clinical meaning, miR-196a was measured in the serum of 105 CC and 86 CIN patients and compared to healthy controls (N = 50). The study results showed that patients with CC had significantly higher levels of serum miR-196a than CIN patients and healthy subjects (*p* < 0.05 and *p* < 0.01, respectively); moreover, the expression of miR-196a significantly correlated with primary tumor features, such as size and grading (*p* < 0.05 in both instances), as well as the presence of lymph node metastases (*p* < 0.05) and clinical stage (*p* = 0.004). Interestingly, the five-year OS rate of women with serum miR-196a levels above the mean value was significantly worse than that of patients in the “lower” group (39.47% versus 73.13%; *p* = 0.004) [72].

Other circulating miRNAs have been suggested as “predictive” of lymph node metastases in early-stage CC patients [70,71,104]; among these, miR-20a, miR-1246, miR-2392, miR-3147, miR-3162-5p, and miR-4484 appeared very promising due to their low susceptibility to variations observed between tissue and serum samples [71].

More recently, a small study by Sun et al. has reported significantly higher serum concentrations of miR-425-5p in CC patients compared to both women with benign cervical disease and healthy controls. In particular, miR-425-5p upregulation has been found to correlate with TNM stage (*p* = 0.0003) and presence of lymph node metastases (*p* = 0.0037), as well as poor OS (*p* = 0.0571) and DFS (*p* = 0.0046) [76], although validation of these data on a wider patient series is mandatory before any clinical application. Indeed, some drawbacks still limit the wide-scale use of miRNAs in the clinical practice, including technical and processing variability among studies, poor specificity and reproducibility, as well as scarcity of prospective validation studies [105,106].

Several deregulated miRNAs have been proposed as putative therapeutic targets; in this regard, miR-486-5p, namely, an activator of the PI3K/Akt pathway via *PTEN*, was found by Li and coworkers to act not only as a reliable CC diagnostic biomarker (AUC = 0.90) in agreement with previous findings [73,82,84] but also as a promising target, as emerged from pre-clinical experiments in which the specific inhibition of miR-486-5p in human CC HeLa cells reduced proliferation, migration, and colony formation potential while impairing tumor formation in mice [82].

On the other hand, several authors tried to restore, through specific lentiviruses or by using miR-mimics, the expression of tumor suppressor miRNAs found downregulated in CC, including miR-30e [77], miR-138 [74], miR-148b [75], and others (Table 3) [78,79,80,81,107]. Interestingly, the upregulation of such epigenetic modulators was found able to inhibit tumor cell proliferation and invasion in vitro, as well as xenograft growth in vivo, but further investigation is still needed to light up the clinical utility of these therapeutic approaches in humans, as well to explore the onset of potential off-target effects.

## 5. Exosomal miRNAs

In addition to the huge number of circulating free RNAs described in CC patients so far, a more recent line of research is focusing on miRNAs shuttled within exosomes, namely, extra-cellular vesicles with a diameter of about 30–150 nm that can be easily detected in body fluids thanks to their abundance and stability [108]; far from being mere cellular “garbage bins”, exosomes actively participate in the crosstalk between cancer cells and tumor microenvironment to control cell proliferation, immune-evasion, and formation of pre-metastatic niches [57,87].

In a study by Zheng et al., exosomal miRNA sequencing was performed in plasma samples from CC and CIN patients, as well as healthy volunteers, to identify DEmiRs as candidate diagnostic biomarkers. Among these, miR-30d-5p and let-7d-3p were validated in 203 plasma samples by using dPCR, presenting an AUC value of 0.922 and 0.828 in the training and validation sets, respectively, together with positive and negative predictive values not inferior to 0.80 [85].

In another recent work, a preliminary plasma exosomal RNA sequencing has been performed in six CC patients and healthy controls, and 39 DEmiRs have been identified between the two populations [86]; among these, miR-125a-5p has been selected due to its previously described role in cancer [109,110] and further quantified in the plasma of 38 women with CC compared to 22 healthy subjects. Interestingly, not only miR-125a-5p levels have been found significantly lower in cancer patients compared to controls (*p* < 0.001), with a diagnostic sensitivity and specificity of 59.1 and 84.2%, respectively, but a clear association has emerged between miR-125a-5p expression and clinical parameters, such as patient age, tumor size, and FIGO stage [86], which may deserve further investigation in prospective studies.

## 6. Conclusions

From a careful analysis of the liquid biopsy approaches used in CC patients, several interesting considerations emerge that, depending on the clinical context, could help in choosing the most appropriate analytical method (Table 4).

As for the quantification of CTCs, the most recent results appear to be very promising in terms of prognostic applications and evaluation of response to therapy, although the identification methods are not standardized yet, as well as there are no data on deep molecular analyses. Alternatively, the identification of somatic gene mutations and sequence variants or gene fusions on ctDNA is to be considered a potentially valuable tool for diagnostic and prognostic purposes, as well as for the identification of novel therapeutic targets. Moreover, ctDNA content has been associated with FIGO stage and metastases, and for these reasons, several scientific societies have proposed extensive use of ctDNA analysis in CC precision medicine programs. Also, the use of high sensitivity dPCR of HPV ctDNA to detect high viral load in the peripheral blood has allowed to recognize patients at high risk of relapse and death within 5 years. However, this approach does not provide information about tumor heterogeneity, which is instead detected by molecular analyses on CTCs.

The most recent evidence in CC liquid biopsy deals with the identification of several RNAs, namely mRNAs, lncRNAs, and miRNAs. HPV and Bmi mRNA are the most commonly detected circulating mRNAs. Both derive from CTCs and are considered markers related to poor DFS and OS. However, the cons in mRNA analysis are due to the presence of ribonucleases in peripheral blood, as well as to contamination by intracellular mRNAs, that limit the reproducibility and applicability of the method.

The combined analysis of lncRNAs or miRNAs looks more promising in this setting.

In conclusion, liquid biopsy leads to several opportunities to make early diagnoses, monitor tumor response to treatment, and detect emerging resistance, through the appearance of new genetic alterations. Moreover, liquid biopsy is a low invasive method, feasible also in social and cultural disadvantaged contexts. However, despite the promising results achieved to date, further large prospective clinical trials are needed to standardize and validate analytical methods, as well as to reach scientific consensus regarding the most sensitive and specific biomarkers to be validated.

## Figures and Tables

**Table 1:** Summary of methods used to identify CTCs in CC.

Method	Markers for CTC Detection	N. of Patients	Clinical Significance	Reference
Reverse transcriptase-polymerase chain reaction (RT-PCR)	Squamous-cell carcinoma (SCC) antigen mRNA	15	Prognostic	[30]
RT-PCR	HPV type 16 E6 mRNA	15	Prognostic	[31]
Nested RT-PCR	CK19 mRNA	84	None	[32]
Digital-direct-RT-PCR	HPV16, HPV18 mRNA	10	None	[33]
Filtration through 8μm membrane pores and in vitro culture of CTCs	Cytomorphological evaluation and gene expression profiling	1	None	[34]
Peripheral blood cell infection with a green fluorescent protein (GFP)-modified adenoviral vector (OBP-1101) and subsequent fluorescence imaging and capture of GFP^+^/CD45^−^ CTC	E6/E7 HPV gene	23	None	[35]
Negative enrichment with anti-CD45 microbeads and fluorescence in situ hybridization (FISH) for CEP8 probe	Hyperdiploid CEP8+/DAPI+/CD45-	99	Prognostic	[36]
CanPatrol^TM^ technique and anti-CD45 antibody	RNA in situ hybridization for epithelial (EPCAM, CK8) and mesenchymal (Vimentin, TWIST) markers	90	Prognostic	[37]
Negative enrichment with anti-CD45 microbeads and FISH for CEP8 probe	Hyperdiploid CEP8^+^/DAPI^+^/CD45^−^	107	Prognostic	[29]
CellSearch system	Pan-CK^+^/CD45^−^	176	Predictive	[38]

**Table 2:** Pre-clinical and clinical studies investigating the role of circulating ctDNA in CC.

Mutated Genes/Viral DNA	Method	N. of CC Patients	Putative Clinical Validity	References
PIK3CA	dPCR	117	Prognostic	[48]
ALK, RET, CSF1R, MET, EGFR, APC, ABL1, NOTCH1, KDR, HNF1A, PDGFRA, ATM, SMO, ERBB2, FGFR2, GNAS, TP53, PTPN11, KRAS, CDH1, FLT3, FGFR3, MLH1, PIK3CA, PTEN, JAK3, MPL, ERBB4, KIT, RB1, IDH1	NGS	57	Prognostic	[49]
ZFHX3, KMT2C, KMT2D, NSD1, ATM, RNF213, FAT1, CHD4, FAT4, TRRAP, EP300, PIK3CA, PTEN, TP53, ARID1A, CTCF, PIK3R1, FXBW7	NGS	24	Follow-up	[50]
PIK3CA, TP53, FXBW7, ERBB2, PTEN, CDKN2A, KRAS, BRAF, MYC, MET, ARID1A, CCNE1, FCFR2, APC, CTNNB1, NRAS, CCND1, TERT	NGS	13	Prognostic, Predictive	[44]
PIK3CA, BRAF, GNA11, FBXW7, CDH1, ALK, STK11, VHL, PDGFRA, HNF1A, MPL, ABL1, RET, KDR, KIT, CDFR1, ATM, EGFR, FGFR1, FGFR2, GNAS, AKT1, KRAS, PTEN, SRC, FLT3, SMO, HRAS, JAK3	NGS	82	Prognostic, Follow-up	[51]
PIK3CA (30.1%), MLL3, TP53, MLL2, EP300, PTEN, FGFR3, DNMT3A, PTCH1, TERT, AKT1, BRAF, BRCA1, ERBB2, TSC2	NGS	126	Prognostic, predictive	[42]

**Table 4:** Pros and Cons of liquid biopsy techniques applied to CC.

Method	Advantages	Disadvantages	Clinical Applications
Circulating tumor cells (CTCs)	Visualization of intact cells for morphologically and immunophenotypical characterization Opportunity for DNA and RNA molecular characterization Opportunity for functional in vitro/in vivo assays	Poor efficiency of isolation from blood Low amount of detectable CTCs Markers used to identify CTCs are often lost during the EMT process Heterogeneity of the CTC populations	CTC count is significantly associated with FIGO tumor stage [39,40,41] CTC count has prognostic meaning and is significantly associated with DFS [36] CTC count may guide treatment selection [29,38]
Circulating tumor DNA (ctDNA)	Easy and well established isolation procedure Higher sensitivity Suitable to identify tumor molecular alterations	Difficulty in discriminating ctDNA from normal cfDNA Extremely low levels of ctDNA Rapidly degraded in plasma, with short half-life Not suitable for functional assays	Specific pathogenic mutations are related to shorter PFS [57], decreased DFS, and OS [52] ctDNA analysis may provide information on disease progression and metastasis onset [54] Mutational analysis may enable evaluation of treatment response over time [46,56]
Circulating Coding RNAs	• Early detection of cancer and disease monitoring	High variability between isolation techniques Lack of standardized protocols RNA instability Potential contamination by intracellular mRNAs	Prognostic markers for lymph node and distant metastases [63] Correlation with FIGO clinical stage [64] Correlation with DFS and OS [61]
Circulating Non-coding RNAs	Potentially detectable in multiple body fluids (plasma, serum, urine, saliva) Stable in blood (compared with other nucleic acids)	High variability between isolation techniques Lack of standardized protocols In some cases microRNA profiles have been inconsistent from study to study	Diagnostic biomarkers [65,70,76,81,85,86,87,90,94,97,98] Prognostic markers for OS [81,90,94,97] Prognostic markers for lymph node metastases [85,95,96,97] Putative therapeutic targets [98,100,101,102,103,104,105,106]
Exosomal miRNAs	Inherent stability (protect from degradation) High sensitivity High serum concentration	High variability between isolation techniques Lack of standardized protocols	• Diagnostic biomarkers [108,109,110]
